# Clinical Validation of Integrated Nucleic Acid and Protein Detection on an Electrochemical Biosensor Array for Urinary Tract Infection Diagnosis

**DOI:** 10.1371/journal.pone.0026846

**Published:** 2011-10-31

**Authors:** Ruchika Mohan, Kathleen E. Mach, Moran Bercovici, Ying Pan, Lakshmi Dhulipala, Pak Kin Wong, Joseph C. Liao

**Affiliations:** 1 Department of Urology and Bio-X Program, Stanford University School of Medicine, Stanford, California, United States of America; 2 Veterans Affairs Palo Alto Health Care System, Palo Alto, California, United States of America; 3 Department of Aerospace and Mechanical Engineering, The University of Arizona, Tucson, Arizona, United States of America; University of Pennsylvania, United States of America

## Abstract

**Background:**

Urinary tract infection (UTI) is a common infection that poses a substantial healthcare burden, yet its definitive diagnosis can be challenging. There is a need for a rapid, sensitive and reliable analytical method that could allow early detection of UTI and reduce unnecessary antibiotics. Pathogen identification along with quantitative detection of lactoferrin, a measure of pyuria, may provide useful information towards the overall diagnosis of UTI. Here, we report an integrated biosensor platform capable of simultaneous pathogen identification and detection of urinary biomarker that could aid the effectiveness of the treatment and clinical management.

**Methodology/Principal Findings:**

The integrated pathogen 16S rRNA and host lactoferrin detection using the biosensor array was performed on 113 clinical urine samples collected from patients at risk for complicated UTI. For pathogen detection, the biosensor used sandwich hybridization of capture and detector oligonucleotides to the target analyte, bacterial 16S rRNA. For detection of the protein biomarker, the biosensor used an analogous electrochemical sandwich assay based on capture and detector antibodies. For this assay, a set of oligonucleotide probes optimized for hybridization at 37°C to facilitate integration with the immunoassay was developed. This probe set targeted common uropathogens including *E. coli*, *P. mirabilis*, *P. aeruginosa* and *Enterococcus* spp. as well as less common uropathogens including *Serratia*, *Providencia*, *Morganella* and *Staphylococcus* spp. The biosensor assay for pathogen detection had a specificity of 97% and a sensitivity of 89%. A significant correlation was found between LTF concentration measured by the biosensor and WBC and leukocyte esterase (p<0.001 for both).

**Conclusion/Significance:**

We successfully demonstrate simultaneous detection of nucleic acid and host immune marker on a single biosensor array in clinical samples. This platform can be used for multiplexed detection of nucleic acid and protein as the next generation of urinary tract infection diagnostics.

## Introduction

Urinary tract infection (UTI) is a common bacterial infection that affects all patient demographics. Diagnostic criteria include presence of urinary symptoms (e.g. frequency, urgency, dysuria), urinalysis showing pyuria, and urine culture showing ≥10^5^ cfu/ml uropathogen. For culture and the associated antimicrobial susceptibility testing (AST), urine samples are sent to a clinical microbiological laboratory, which has a typical delay of 2–3 days. Due to this delay, physicians often prescribe antibiotics empirically based on symptoms and historic antimicrobial susceptibility data. While empiric treatment is sufficient in many patients, a more complete diagnosis is beneficial for patients with recurrent, complicated UTI such as those dependent on urinary catheters for bladder emptying. Catheterized patients are prone to bacterial colonization in the bladder that may not necessitate treatment (i.e. asymptomatic bacteriuria) but are also at a greater risk of infection with resistant pathogens due to frequent exposure to antibiotics [1].

As a part of the host innate immune response, white blood cells (WBCs) are recruited to the urinary tract in response to the presence of bacterial pathogens. Typically, urinary WBC counts are determined by urinalysis in a centralized laboratory or approximated by a dipstick test at the point of care. Although identification of pathogens gives useful information for diagnosis of UTI, it does not distinguish colonization from infection, determine severity of infection, or the degree of host response. Quantitative detection of urinary proteins indicative of host immune response, in addition to pathogen identification, provides a more comprehensive diagnosis of UTI and a significant advancement towards a personalized medicine for UTI treatment [2].

There is significant interest to develop biosensor technology for applications in healthcare, environmental, and food safety monitoring [3], [4]. Previously, we developed an electrochemical biosensor for UTI diagnostics. This biosensor array consists of 16 individually addressable sensors that can be functionalized with oligonucleotide probes or antibodies for detection of urinary nucleic acids or proteins, respectively [5], [6]. The detection strategy is based on a sandwich assay, coupled to an HRP based redox reaction, giving rise to quantifiable electrical signal.

**Figure 1:**
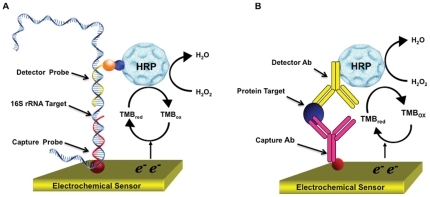
Schematics of urine-based diagnostics for electrochemical biosensor detection of nucleic acids and proteins. (A) Schematic of pathogen identification based on sandwich hybridization of bacterial 16S rRNA with capture and detector oligonucleotide probes; (B) Schematic of immunoassay based on sandwich detection host urinary protein with capture and detector antibodies. The two assays share similar assay parameters, including surface functionalization with biotinylated capture probes/antibodies, probe-target binding at 37°C, and amperometric detection using horseradish peroxidase (HRP) as the signaling enzyme [7], [8].

Using the electrochemical biosensor, we demonstrated a 1-hour biosensor assay for detection of pathogen 16S rRNA from patient urine samples using the biosensor [7]. An advantage of this electrochemical sensor platform is that it can be adopted for both nucleic acids and protein detection. We have also developed an immunoassay using the biosensor to detect lactoferrin (LTF), an iron binding protein secreted by WBCs as part of innate immune response [8]. Here we report an integrated biosensor assay for simultaneous detection of nucleic acid and protein targets for UTI diagnosis (Figure 1). We modified and expanded the panel of oligonucleotide probes to target additional uropathogens and optimized binding parameters for simultaneous pathogen and protein detection. The integrated pathogen 16S rRNA and host LTF assay was performed in 113 clinical urine samples collected from patients at risk for complicated UTI. Data from the integrated biosensor assay was compared with clinical laboratory results and correlated with patient demographics.

## Results

### Development of oligonucleotide probe pairs optimized for hybridization at 37°C

**Figure 2:**
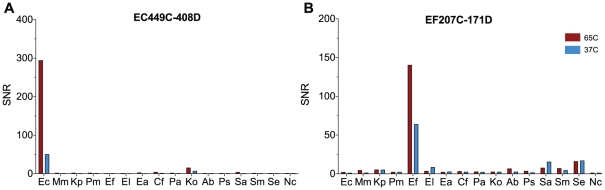
Comparison of biosensor pathogen detection using 35-bp probes at 65 and 37°C. Probe selectivity and sensitivity was tested against different uropathogens. A. The biosensor signal for detection of *E. coli* with EC449C-EC08D was 1996 nA at 65°C compared to 1270 nA at 37°C and this probe set showed non-specific binding with *K. oxytoca* at both temperatures. B. The biosensor signal for detection of *E. faecalis* with EF207C-EF171D was 1107 nA at 65°C and 549 nA at 37°C and this probe set showed non-specific binding with *K. pneunomiae*, *E. cloacae*, *S. aureus*, *S. epidermidis*. The bacterial species are abbreviated on the X-axis with capital letter for genus and small letter for species.

In our previous work, the biosensor assays for nucleic acids and protein detection were based on target binding of nucleic acids at 65°C [5], [6], [7] and protein biomarkers at 37°C [8]. In an effort to integrate the two assays on a single sensor array under uniform target binding conditions (i.e. 37°C), we examined whether the oligonucleotide probe pairs previously developed for hybridization at 65°C can provide adequate signal strength and specificity at 37°C. Figure 2 shows representative experiments comparing the specificity of the our previously reported probe pairs targeting *E. coli* (EC449C-408D) and *Enterococcus* spp. (EF207C-171D) at 37°C and 65°C hybridization. Overall, decreased signals and higher cross-reactivity with other non-specific pathogens were observed at 37°C. Similar decrease in performance was observed with the 5 other probe pairs at 37°C (data not shown). Based on these findings, we set out to develop a new set of probes optimized for hybridization at 37°C to facilitate integration with the immunoassays.

A systematic approach of combining *in silico* predictions and biosensor experiments was used to design and validate a panel of new capture and detector probe pairs against bacterial 16S rRNA. We selected 20–24 bp as the probe length for hybridization at 37°C, which is shorter than our previous probe pairs designed for hybridization at 65°C [5], [7], and longer than probes reported by others for hybridization at room temperature [9]. Primrose [10] software, in conjunction with the probe match function of Ribosomal Database Project (RDP) [11], was used to identify candidate capture probe sequences. For *E. coli*, 34 candidate probe sequences were identified. To further narrow the selection, Geneious sequence alignment software (Biomatters Inc, New Zealand) [12] was used to match the candidate probes with a database of 16S rRNA gene sequences from 133 uropathogenic bacteria (three or more sequences of each common uropathogen). For probes designed to target *E. coli*, this reduced the candidate probe pairs from 34 to 5. Detector probes consisting of the flanking sequence immediately upstream to the capture were designed as previous work demonstrated that contiguous capture and detector probes provide the best signal to noise ratio (SNR) [6].

**Figure 3:**
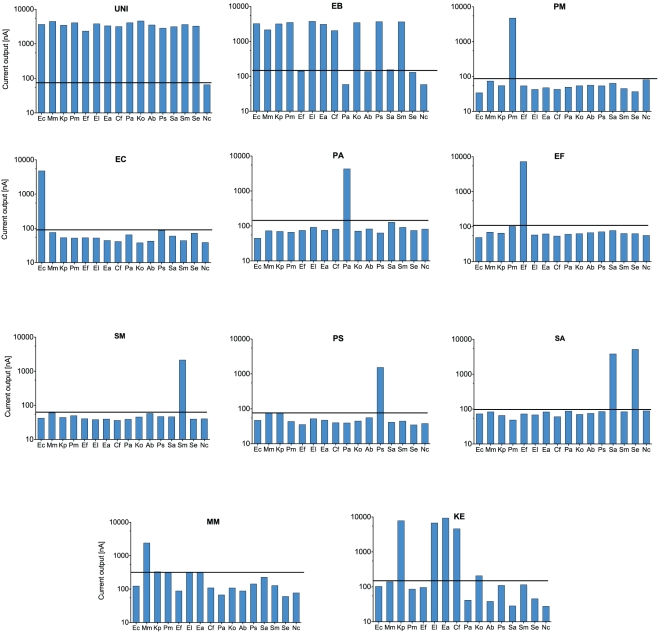
Specificity of biosensor pathogen detection with 20–24 bp oligonucleotide probes designed for hybridization at 37°C. Log signal intensities of current (nA) are plotted on the Y-axis. Common uropathogens tested are indicated on the X-axis. The line over the bars indicates the threshold for positive biosensor signal 3SD (log_10_ unit) over the negative control for UNI, EB, PM, EC, PA, EF, SM, PS, SA and 5SD (log_10_ unit) over negative control for MM and KE probes. Consistent with their *in silico* design, UNI probe pair detected all bacterial species and EB probe pair detected members of *Enterobacteriaceae*. PM, EC, PA, EF, SM, PS, SA and MM probe pairs specifically detected *Proteus mirabilis*, *E. coli*, *Pseudomonas aeruginosa*, *Enterococcus* spp., *Serratia marcescens*, *Providencia* spp., *Staphylococcus* spp, and *Morganella morganii*, respectively. The KE probe pair detected *Klebsiella pneumoniae*, *Klebsiella oxytoca*, *Citrobacter freundii*, *Enterobacter aerogenes* and *Enterobacter cloacae*. Probe sequences are provided in Table 1.

After the *in silico* selection process, 2–5 of the most promising probe pairs for each pathogen were tested using the electrochemical biosensor assay to determine their detection specificity and sensitivity. Each of the probe pairs was tested against 15 different uropathogens to determine their specificity. For example, 2 pairs of *E. coli* probes were tested using the biosensor assay and the probe pair EC471C-447D was selected for the clinical study since it gave the best specificity and SNR. The probe pairs that gave the optimal specificity and the best SNR are shown in Figure 3.

Table 1 shows the sequences of the capture and detector probe pairs and their target pathogen(s). The threshold for positive detection was defined as 3 standard deviations above the negative control for the UNI, EB, EC, PA, EF, SM, PS and SA probe pairs. The universal (UNI) probe pair, which targets a highly conserved region of 16S bacterial rRNA, detected all pathogens tested. The EB probe pair targets the *Enterobacteriaceae* family, which are gram-negative enteric bacteria that constitute majority of the gram-negative uropathogens. Probe pairs targeting *Proteus mirabilis* (PM), *E. coli* (EC), *Pseudomonas aeruginosa* (PA), *Enterococcus* spp. (EF), *Serratia marcescens* (SM), *Providencia* (PS) and *Staphylococcus* spp. (SA) showed specific detection of the respective species. For the MM and KE probe pairs, the threshold for positive detection was defined as 5 standard deviations above negative control in order to maintain specificity, with minor compromise of sensitivity. Using this threshold, the MM probe provides specific detection of *Morganella morganii*. Given the similarity in the 16S rRNA sequences for the *Klebsiella*, *Enterobacter*, and *Citrobacter* spp., KE probe pair has specificity for *Klebsiella pneumoniae*, *Klebsiella oxytoca*, *Enterobacter cloacae*, *Enterobacter aerogenes* and *Citrobacter freundii*.

**Table 1:** Sequences of the oligonucleotide probes used in this study.

Probe pairs (Length in bp)	Sequence (5′-3′)	Species detected
UNI798C (22)	TCGTTTACRGCGTGGACTACCA	Ec, Mm, Kp, Pm, El, Ea, Cf, Cb, Ko, Ps, Sm, Sf, Pr
UNI776D (22)	GGGTATCTAATCCTGTTTGCTC	Pa, Ef, Ee Sa, Se, Ss, Pv, Kz, Xm, Dp, Ab, Af, Fi
EB1275C (23)	ACTTTATGAGGTCCGCTTGCTCT	Ec, Mm, Kp, Kz, Pm, El, Ea, Cf, Ko, Ps, Sm, Pr, Sf,
EB1252D (23)	CGCGAGGTCGCCTTCCTTTGTAT	Cb
PM1019C (22)	AGCGTTCCCGAAGGCACTCCTC	Pm, Pv
PM997D (22)	TATCTCTAAAGGATTCGCTGGA	
EC471C (24)	CTGCGGGTAACGTCAATGAGCAAA	Ec
EC447D (24)	GGTATTAACTTTACTCCCTTCCTC	
PA594C (23)	CCCGGGGATTTCACATCCAACTT	Pa
PA570D (23)	GCTGAACCACCTACGCGCGCTTT	
EF220C (20)	ACCGCGGGTCCATCCATCAG	Ef, Ee
EF200D (20)	CGACACCCGAAAGCGCCTTT	
SM472C (22)	TGCGAGTAACGTCAATTGATGA	Sm
SM450D (22)	RCGTATTAAGYTCACCACCTTC	
PS151C (24)	CCGAAGGTCCCCTGCTTTGCTCCT	Ps, Pr
PS127D (24)	AAGAGATTATGCGGTATTAGCCAC	
SA91C (22)	CCCGTCCGCCGCTAACRTCAGA	Sa, Se, Ss
SA69D (22)	GRAGCAAGCTYCTCGTCYGTTC	
MM181C (22)	GGCGCGAGGCCCGGAGGTCCCC	Mm
MM147D (22)	CGCTTTGGTCCGAAGACATTAT	
KE468C (22)	AGTAACGTCAATCRCYAAGGTT	Ea, El, Kp, Cf, Ko, Kz, Cb
KE446D (22)	ATTAACCTTAACGCCTTCCTCC	

The capture (denoted by “C”) and detector (denoted by “D”) probes were modified with 5′ biotin and 3′ fluorescein, respectively. The degenerate bases R represents bases A or G and Y represents C or T. *E. coli* (Ec), M. morganii (Mm), *K. pneumoniae* (Kp), *K. ozaenae* (Kz), *P. mirabilis* (Pm), *P.vulgaris* (Pv), *E. faecalis* (Ef), *E. faecium* (Ee), *E. cloacae* (El), *E. aerogenes* (Ea), *C. freundii* (Cf), *C. braackii* (Cb), *P. aeruginosa* (Pa), *K. oxytoca* (Ko), *A. baumannii* (Ab), *P. stuarti*i (Ps), *P. rettgeri* (Pr), *S. aureus* (Sa), *S. marcescens* (Sm), *S. fonticola* (Sf), *S. epidermidis* (Se), *S. saprophyticus* (Ss), *X. maltophilia* (Xm), *Diphtheroids* (Dp), *A. baumannii* (Ab), *A. faecalis* (Af), *F. indolgenes* (Fi) were tested against above probe sets.

The limit of detection (LOD) of the biosensor assay was 10^4^ cfu/ml from bacterial culture and clinical urine samples. Similar to our previous results [5], [6], [7] urine samples have the similar LOD as bacterial culture. The molar sensitivity of the assay can be estimated through the number of 16S rRNA copies per bacterial cell. This concentration reportedly varies between 7,000 and 70,000 ribosomes/cell [13], [14] depending on the bacterial species and its growth stage at the time of lysis. We thus estimate the limit of detection of the sensor between 2–20 pM.

### Patient and sample characteristics

In order to validate the new probe pairs in an integrated biosensor assay combining pathogenic nucleic acids and host protein biomarker detection, we used clinical urine samples collected from spinal cord injury (SCI) patients, who are at significant risks of developing complicated, polymicrobial urinary tract infections due to neurogenic bladder and frequent use of catheters. From 111 patients recruited (109 male, mean age 59±13), 113 samples were analyzed, 85 from outpatients and 28 from inpatients. In our study population, 27 samples were collected through spontaneous voiding, 85 samples from catheters, and 1 sample from a patient with ileovesicostomy. Of the 85 catheterized samples, 21 were from standard indwelling Foley catheter, 21 from clean intermittent catheterization, 11 from suprapubic (SP) catheter, and 32 from condom (C) catheter.

Table 2 shows the characteristics of the urine samples as reported by the clinical microbiology laboratory. Positive urine culture was found in 79 samples, out of which 22 contained a single species, 13 had two or three bacterial species and 44 samples were reported as mixed urogenital flora. Similar to our prior observations [7], *E. coli* was the most common pathogen followed by *K. pneumoniae*. *P. aeruginosa* was the most common pathogen found in samples containing two species.

**Table 2:** Sample characteristics.

	Number of samples
**No growth**	**34**
**Single species**	**22**
*E. coli*	9
*K. pneumoniae*	4
*Enterococcus spp.*	2
*P. mirabilis*	2
*P. aeruginosa*	2
*M. morganii*	1
*C. koseri*	1
*E. aerogenes*	1
**2–3 species**	**13**
*E. coli*, *E. cloacae*	1
*E. coli*, *S. agalactiae*	1
*P. aeruginosa*, *Enterococcus* spp.	2
*P. aeruginosa*, *A. baumannii*	1
*P. aeruginosa*, *S. aureus*	2
*P. aeruginosa*, *K. pneumoniae*	1
*S. marcescens*, *S. agalactiae*	1
*Enterococcus spp.*, *K. pneumoniae*	1
*K. pneumoniae*, Lactose-Neg Gram-Neg Rod	1
*P. aeruginosa*, *P. stuartii*, *S. aureus*	1
*P. mirabilis*, *Enterococcus* spp.	1
**Mixed urogenital flora**	**44**

### Clinical validation using the integrated biosensor array

**Figure 4:**
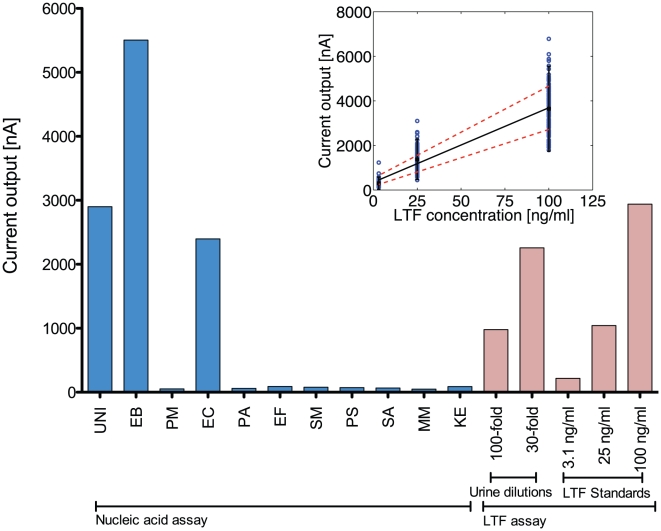
Example of integrated bacterial 16S rRNA and LTF biosensor assay from a urine specimen. Each array consisted of 16 electrochemical biosensors. In this study, eleven sensors were used for nucleic acid assay for pathogen identification and 5 sensors for immunoassay for LTF detection. For measurement of LTF directly from urine, two urine dilutions were tested and the concentration of LTF was determined using the standard curve generated from three known concentrations of LTF. For this urine sample, positive signals from the UNI, EB and EC probes indicated this sample contained *E. coli* and the measured LTF concentration of 2106 ng/ml indicated significant pyuria. This interpretation was confirmed by the clinical microbiology laboratory, which determined the sample contained >100,000 cfu/ml *E. coli* and >100 WBC/HPF. Inset shows the standard curve for detection of LTF based on 123 measurements on different biosensors on different days. The results show good reproducibility and could potentially be used for detecting the concentration of LTF from urine samples without the need of a standard curve on each biosensor.

The collected urine samples were tested using the integrated biosensor array for quantitative detection of pathogen(s) and LTF. Each urine sample was tested with a single 16-sensor array. The sensors were functionalized with the new panel of the capture probes against 16S rRNA and the capture antibody against LTF. Both hybridization and the antibody-antigen binding steps were performed at 37°C. Figure 4 shows the output from the integrated biosensor assay for analysis of a patient urine sample. The biosensor results were compared with clinical microbiology laboratory results for culture and urinalysis. Consistent with the clinical microbiology report, the biosensor assay identified the presence of *E. coli* and significant pyuria.

Of the 34 samples that were culture negative, biosensor results agreed with clinical microbiology in all but one sample, yielding a specificity of 97%. In this particular sample, the clinical microbiology laboratory reported it to be culture negative, while independent culturing in our laboratory indicated 4×10^7^ cfu/ml bacteria. The clinical microbiology laboratory reported 79 samples as culture positive, out of which biosensor detected 70 as positive, yielding a sensitivity of 89%. The samples that were not detected by the biosensor had bacterial concentrations ranging from 10^3^ to 10^4^ cfu/ml, which was at or below the LOD of the biosensor assay.

The biosensor assay detected bacteria in all 22 urine samples containing a single uropathogen and further identified the bacterial species in 16 of these samples and the family (e.g. *Enterobacteriaceae*) in 6 samples, with results in agreement with the clinical microbiology results. Samples that were identified to the family level included the samples containing *K. pneumoniae* and *E. aerogenes*, which were targeted by the KE, EB, and UNI probes on the biosensor.

The majority of the samples with polymicrobial infection were reported as ‘mixed flora’ and not speciated by the clinical microbiology laboratory, thus precluding direct comparison with the biosensor results at the species level. Out of the 44 ‘mixed’ samples identified by the clinical microbiology laboratory, the biosensor detected 37 of these samples as positive by the UNI probe. The bacterial concentration in four of the missed samples was below the biosensor's limit of detection.

Of the 13 polymicrobial samples that were speciated, the biosensor accurately identified all species in four of these samples and at least one of the species in six samples. The samples missed by the biosensor either had pathogens below the limit of the detection of the biosensor or specific probes for the pathogens were not available.

LTF concentration was determined in each urine sample along with the detection of pathogens by 16S rRNA in the integrated biosensor assay. Data from the integrated biosensor assay was correlated with results obtained from urinalysis. Spearman's correlation coefficient was used to evaluate relationship between different clinical parameters, bacterial and LTF concentration. Consistent with our previous report [8], we found a significant correlation between LTF concentration measured by the biosensor and WBC (ρ = 0.56, p<0.001) and leukocyte esterase (ρ = 0.76, p<0.001) reported by urinalysis. A significant correlation was also found between bacterial concentration and LTF concentration (ρ = 0.61, p<0.001), WBC (ρ = 0.53, p<0.001) and leukocyte esterase (ρ = 0.67, p<0.001 for each). Similar correlations between bacterial concentrations and WBC have been reported previously [15], [16], [17].

## Discussion

System integration is one of the most challenging aspects of developing point-of-care diagnostics [18], [19]. One of the goals of the current study is integrated detection of two different targets, bacterial nucleic acids (i.e. 16S rRNA) and host protein (i.e. LTF) onto a single biosensor platform. We present clinical validation of an electrochemical biosensor array for UTI diagnosis capable of pathogen identification by detection of bacterial 16S rRNA and assessment of pyuria by detection of LTF. While our previous work demonstrated independent biosensor assays for pathogen identification and LTF [5], [7], [8], here we have modified the assay to allow integration of the assays on a single biosensor array at 37°C without loss of sensitivity or specificity.

For this study, we developed new panel of oligonucleotide probes for pathogen identification that facilitate integration with synchronous protein detection under common assay conditions. While nucleic acid detection works well at higher temperature [5], [7], protein detection may be compromised at high temperatures due to decreased stability or aggregation of proteins [20]. In our earlier studies, oligonucleotide probes designed for hybridization at 65°C were used for detection of bacterial 16S rRNA. These probes showed reduced sensitivity and specificity at 37°C.

We have expanded our probe panels to target additional uropathogens including *Serratia*, *Providencia*, *Morganella* and *Staphylococcus*, which represents significant progress towards development of a comprehensive panel for detection of clinically significant uropathogens. The shorter probe length of 20–24 bp allowed targeting a greater number of regions of sequence diversity within 16S rRNA. The lower hybridization temperature not only facilitates integration of the nucleic acid assays and immunoassays, but will also be useful for further integration with a biosensor-based antimicrobial susceptibility test, which is also done at 37°C [21].

We successfully demonstrated simultaneous detection of nucleic acid and host immune marker on a single biosensor array in clinical samples. The two electrochemical assays share similar protocol parameters, including surface functionalization with biotinylated capture probes/antibodies, probe-target binding at 37°C, and amperometric detection using horseradish peroxidase (HRP) as the signaling enzyme. Furthermore, integration improves the efficiency and reduces the potential human errors associated with running two separate assays. One sample reported to be culture negative, was found to have significant pyuria (75–100 WBC/HPF) and bacteria by microscopy in separate clinical laboratory urinalysis. In our integrated assay, we found this sample to be biosensor positive for UNI, EB and KE probes and contained 9950 ng/ml of LTF. Independent plating in our laboratory further corroborated pathogen identification, indicating the presence of 4×10^7^ cfu/ml bacteria.

LTF is a molecular marker of pyuria [8], [22], and urinalysis measurement of pyuria is routinely performed as part of UTI diagnosis. Previous studies have suggested LTF as a promising biomarker for UTI diagnosis [2], [22]. Given the relative abundance in urine, LTF also serves as a suitable target for new assay development using the electrochemical biosensor. In this study we examined the utility of measuring LTF in urine samples of spinal cord injury (SCI) patients. These patients are at risk for complicated UTI, due to structural and physiological impairment of bladder emptying, vesicoureteral reflux, and need of indwelling catheters [23]. In this study, the LTF concentration was highly correlated with WBC concentration and leukocyte esterase. While the analytical performance of the integrated assay was promising, we found that LTF level was not predictive of the need for treatment. This is likely due to the association of asymptomatic pyuria associated with urinary catheters, which is common in SCI patients [23], [24]. Future directions include validation of the integrated assay in non-catheterized patients and identification of biomarkers specific to infection in catheterized patients. Our platform will enable efficient integration of additional biomarkers with pathogen identification.

Rapid molecular diagnosis of UTI represents a significant advance in the management of UTI and potentially reduce the practice of prescribing unnecessary antibiotics [25]. We have developed a biosensor platform that enables multiplexed detection of bacterial-specific nucleic acids and host immune response protein, and demonstrated its validity in clinical samples. Efforts are underway to improve the detection sensitivity of the assay by incorporating electrokinetic manipulation on the biosensor platform [26]. In the future, we intend to utilize this capability and explore multiplexed detection of additional host immune response biomarkers in order to better differentiate bacteriuria from urinary tract infections.

## Methods

### Ethics statement

Patient urine samples were collected with approval from Stanford University's Institutional Review Board (IRB) and Veterans Affairs Palo Alto Health Care System's (VAPAHCS) Research and Development committee. Oral informed consents were obtained from all study subjects due to spinal cord related physical impairment and low risk nature of the study. Oral consent was approved by both Stanford IRB and VAPAHCS R&D committee and was documented with a written record of participants name, date of birth, medical record number and date of sample collection, which served as both the record of oral consent and enrollment record.

### Design of oligonucleotide probes for pathogen detection

Oligonucleotide probes targeting the 16S rRNA of uropathogens were designed using Primrose v2.17 and RDP Release 8. The selectivity of the potential probes identified by Primrose was assessed using the probe match function of RDP Release 10. Probe selectivity was further verified by alignment against 16S rDNA sequences from bacterial isolates from urine (at least 3 sequences for each species) using Geneious. 16S rRNA gene sequences from bacterial isolates were kindly provided by David Haake (unpublished data).

Oligonucleotide probes are designated as capital letters to detect all Eubacteria (UNI), *P. mirabilis* (PM), *E. coli* (EC), *P. aeruginosa* (PA), *Enterococcus* spp. (EF), *S. marcescens* (SM), *Providencia* spp., (PS), *M. morganii* (MM), *Staphylococcus* spp. (SA), *K. pneumoniae*, *K. oxytoca*, *E. cloacae*, *E. aerogenes*, *C. freundii* (KE). UNI and EF probe pairs were designed from the existing probe-pairs for hybridization at 65°C [5]. The probe pair targeting *Enterobacteriaceae* (EB) family was previously described [9]. The oligonucleotide probe pairs (20–24 bp) were synthesized by Integrated DNA Technologies Inc (San Diego, CA).

### Bacterial strains and cultivation

To validate their selectivity, oligonucleotide probes were tested against uropathogen isolates from patient urine samples obtained at VAPAHCS as well as strains obtained from American Type Culture Collection (ATCC): *C. freundii* 8090, *Enterococcus faecalis* 49532, *P. aeruginosa* 10145, *E. aerogenes* 13048, *E. cloacae* 13047, *K. oxytoca* 49131, and *A. baumannii* 19606. Uropathogen isolates from patient samples were identified by clinical microbiology laboratory analysis and confirmed by 16S rRNA gene sequencing (Sequetech, USA) using the primers 8UA/907B and 774A/1485B [27]. Bacterial strains were grown in Luria Bertani broth to logarithmic phase as measured by OD_600_. The bacterial concentration was determined by serial dilution and plating. Bacterial cell pellets containing 10^8^ cfu were used for probe validation.

### Biosensor assay for probe validation

The electrochemical biosensor array (GeneFluidics, USA) surface was functionalized as described previously [5], [6], with the following modifications: EZ-link Amine-PEG2-Biotin (Pierce, Rockford, IL) concentration was 0.5 mg/ml in 0.1 M phosphate buffer and the streptavidin (Sigma-Aldrich, St. Louis, MO) concentration was 0.05 mg/ml in phosphate buffer containing 2.5% BSA. Six µl of the reagents and analytes were spotted on the sensor. Sample preparation, sandwich hybridization of oligonucleotide probes against bacterial 16S rRNA and amperometric detection were performed as previously described [6], with the exception that the hybridization steps were performed at 37°C. Phosphate buffer (1 M, pH 7.2) containing 2.5% BSA with the detector probe was used as a negative control.

### Study participants and clinical samples

Urine samples were collected from the VAPAHCS spinal cord injury unit between July 2009 and April 2010. The decision to collect urine was based on clinical judgment of the treating physician (patients suspected of a UTI or as part of their routine care). Depending on the patient's bladder emptying status, urine samples were collected from an indwelling catheter, straight catheterization, or voiding. For each sample, one aliquot was used for the biosensor experiment and the second was sent to clinical laboratory for urinalysis (white blood cells per high power field (WBC/HPF), pH, specific gravity, leukocyte esterase, nitrite) and culture and susceptibility. Qualitative and quantitative plating was done in our laboratory on BBL CHROMagar™ Orientation and TSA with 5% sheep blood (BD Diagnostics, Sparks, MD) and LB media, respectively. For biosensor pathogen identification assay, 1.5 ml of the sample was centrifuged and the pelleted fraction was stored. Whole urine was stored for the LTF biosensor immunoassay. The samples were stored at −80°C until tested.

### Integrated nucleic acid and protein biosensor assay

For the integrated assay, the surface of 11 of the 16 sensors on the array was functionalized with the capture probes for pathogen identification and 5 sensors were functionalized with capture antibodies for LTF detection (rabbit biotinylated polyclonal anti-LTF, ab25811, Abcam). The biosensor with the capture probes and antibody was incubated at 37°C for 30 min. The urine pellet was lysed as previously described [5] with the addition of 0.25 mg/ml of lysostaphin (Sigma, St Louis, MO) to the lysis buffer. The lysate was neutralized with 50 µl of phosphate buffer containing 2.5% BSA and divided to 11 aliquots of 6 µl each. Different detector probes [1 µl of 0.5 µM in phosphate buffer (pH 7.2) with 2.5% BSA] were added to each aliquot and incubated at 37°C for 15 min for hybridization of the bacterial 16S rRNA to the detector probe. The detector probe-16S rRNA complex was deposited on sensors containing the corresponding capture probe. For LTF detection, LTF standards at 100, 25, 3.1 ng/ml and 100× and 30× diluted urine samples were deposited on electrodes with detector antibody-LTF complex. Biosensor assay was completed as described previously [8].

### Data analysis

Clinical microbiology results were used as the standard to determine the diagnostic sensitivity and specificity of the biosensor assay for pathogen identification. Per VAPAHCS clinical microbiology laboratory protocol, samples containing bacterial concentrations of 10,000 cfu/ml or less were not speciated. In addition, polymicrobial samples containing 3 or more species were typically not speciated and reported as mixed flora. Similar to our previous report, signal from UNI probe was used to calculate the diagnostic sensitivity of the biosensor [7]. Biosensor signals were log_10_ transformed and positive signals were defined as greater than 3 SD (log_10_ units) over negative control for UNI, EB, PM, EC, PA, EF, SM PS and SA probes and greater than 5 SD (log_10_ units) over negative control for MM and KE probes. The lowest signal on the biosensor assay was used as the negative control.

The urinary LTF concentrations were compared with results obtained from urinalysis (WBC/HPF and leukocyte esterase) to assess validity of the biosensor. To facilitate analysis, WBC/HPF obtained from urinalysis were divided into four groups (0–2, 3–10, 11–50, >50). Samples containing <2 WBC/HPF were considered negative for pyuria. Descriptive analyses and ranked medians among categorical groups were conducted using Kruskal-Wallis test with GraphPad Prism version 5.0b. Spearman correlations were performed using SAS version 9.1.3 to assess ranked correlation between clinical and biological variables.
